# Breaking institutional barriers to enhance women’s participation in and benefit from the Peste des Petits Ruminants and Newcastle Disease vaccine value chains for Sembabule district of Uganda

**DOI:** 10.1371/journal.pone.0270518

**Published:** 2022-10-13

**Authors:** Winnie Bikaako, Patricia Kabahango, Kenneth Mugabi, Agnes Yawe, Kisembo Stallon, Elizabeth Kyewalabye, Lillian Tukahirwa, Dean Kusiimakwe, Meghan Stanley, Beth Miller, Anthony Mugisha, Marieke H. Rosenbaum, Hellen Amuguni

**Affiliations:** 1 Africa One Health University Network (AFROHUN), Kampala, Uganda; 2 College of Veterinary Medicine, Animal Resources and Bio-security, Makerere University, Kampala, Uganda; 3 Department of Animal Health, Ministry of Agriculture, Animal Industry and Fisheries, Kampala, Uganda; 4 Uganda Martyrs University, Kampala, Uganda; 5 Cummings School of Veterinary Medicine, Tufts University, North Grafton, Massachusetts, United States of America; 6 Miller Consulting Inc, Little Rock, Arkansas, United States of America; West Bengal University of Animal and Fishery Sciences, INDIA

## Abstract

This paper describes the institutional context that shapes the visibility and positioning of women along the Peste des Petits Ruminants (PPR) and Newcastle Disease (ND) vaccine value chains for Sembabule district of Uganda. It examines the institutional barriers and opportunities that affect women’s empowerment derived from inclusion of women in the decision-making processes along the livestock vaccine value chain (LVVC) and that can support viable women-centered and owned enterprises, at the vaccine development, delivery, distribution and use level. Qualitative data analysis tools such as focus group discussions, focus meals, jar voices and key informant interviews were used. Using outcome mapping, a stakeholder analysis of the critical partners in the PPR and ND value chain was done involving the regulators, vaccine manufacturers, importers, distributors, agrovets, public and private veterinary service deliverers, local leaders, women groups, and farmers. The study concluded that training related to gender equality and livestock vaccines, infrastructural and technical support to the poultry and goat women and men farmers and other chain actors are inadequate in themselves to increase vaccine adoption and improve livestock productivity in Sembabule district. Strategies that promote gender-transformative collaborative efforts among the LVVC actors and build viable gender-transformed women groups and networks are critical to increase women’s participation in and benefit from the livestock vaccine value chain.

## Introduction

Women comprise approximately 70% of the world’s poor [1]. Although statistics vary drastically all over the world, there seems to be agreement about women constituting a greater proportion of the world’s poor livestock keepers [2]. Poor rural women livestock keepers in low- and middle-income countries (LMIC) are more likely to own chickens and goats. The chickens and goats are critical assets for women to generate income, provide high-protein food for the family, accumulate wealth, confer social status, provide protection against economic shocks and are a financial reserve for the family [1, 3–5]. In many instances, the income generated, although small, goes directly to the women [1]. Village chickens (local breeds), in particular, are generally owned and managed by women and children and are often essential assets for female-headed households [6, 7]. They are also more easily managed and place few or no additional burdens on women as, apart from the provision of household food scraps, the birds find their own feed and require little supervision [1, 8]. Among the smallholder farmers in developing countries, the poorest tend to own only poultry, and as they progress up the ladder out of poverty, pigs and small ruminants (goats or sheep) are next, with cattle, camels or buffalo the most desirable animals [1].

The potential for livestock to improve livelihoods, food security, incomes for poor households, and enhance women empowerment is significant and can only be reached if disease is controlled. Peste des Petits Ruminants (PPR) and Newcastle Disease (ND) have had devastating impacts on small ruminants and poultry in Africa respectively, thereby affecting livestock productivity and livelihoods among rural smallholder farmers, especially women [9]. Empowering female farmers, especially rural subsistence farmers, has been shown to be an effective means of fighting household hunger and poverty and gender equality remains a top developmental priority in the 2015 United Nations Sustainable Development Goals [5]. Studies have shown that female livestock keepers tend to own more small ruminants (goats, sheep, etc.) and poultry than large livestock (water buffalo, cows, etc. [10, 11]. As a result of this differential ownership, women may be more affected by small ruminant and poultry diseases like NCD and PPR. There are also a number of general barriers and constraints that female livestock keepers face that may exacerbate the effects of these diseases on their livelihood. Women have limited access to services, credit, technology, training and information regarding livestock putting them at greater risk of livestock loss [12]. They tend to contribute to the reproductive economy more than their male counterparts and therefore have less access to markets and cooperatives and income generated from livestock [13]. The PPR disease is considered the most significant economic threat to sheep and goat production in LMIC, particularly in Africa and Asia [14]. Despite some serological evidence of PPR virus incursions into Uganda and Kenya in the 1980s and early 2000s, and an isolated report of an outbreak in Uganda in 2003, it was not until 2006–2008 when the first official and large PPR outbreak was reported in the bordering Karamoja region in Uganda. Since then, several PPR outbreaks have been reported in different regions of East Africa, suggesting PPR virus persistence in some nomadic and semi-nomadic pastoralist systems in Western, Northeastern Northern and Central Uganda [15, 16].

An infectious and virulent viral disease, ND causes high mortality in poultry and constrains availability of income and affordable source of animal protein to the rural poor woman. It is endemic in Africa [4] and outbreaks in susceptible flocks regularly result in mortalities of 50% to 100% [8]. In Uganda, the first documented evidence of ND occurred in 1955 in the Central region and the most virulent strain characterised in 1986 was 100% morbidity and 98% mortality [17].

Both PPR and ND can be effectively controlled through vaccination [1, 4, 8, 9, 17]. Livestock vaccines are essential to help prevent and control livestock diseases, prevent the transmission of zoonotic infections to humans, enhance animal sourced food quality, and ultimately contribute to sustainable livelihoods [3]. Livestock vaccination has both direct effects on livestock health and productivity outcomes and indirect effects on household expenditures, such as child education, food, and health care [5]. These indirect effects tend to be gendered. Frequently, vaccines do not reach and thus are not often used by smallholder farmers [1]. Vaccination decreases chicken mortality and results in increased flock size in smallholder production systems, but despite this, the vaccination rate outside of the commercial sector remains low [4]. Adoption of livestock vaccines by smallholder livestock farmers is largely dependent on vaccine availability, vaccine access and vaccine demand [1]. Mutua et al cites several gendered barriers in Kenya and Uganda including insufficient vaccine supply, cost, lack of information by farmers and lack of consent from farmers to have their livestock vaccinated contribute to low vaccine coverage [18]. Donadeu et. al. notes that on the poverty pyramid, the farmer at the bottom usually owns only chicken followed by small ruminants (goats and sheep) and pigs [1]. There is a tendency by governments and other non-government actors to ignore the needs of these marginalized populations, including failure to provide vaccines for their stock. As a result, there are no significant vaccination activities involving chickens and goats which has taken place in Sembabule district, yet this could be the only means of preventing sickness and death of small holder farmers livestock.

To understand the dynamics around vaccine adoption among the women smallholder farmers, this study examines the pathways of the livestock vaccine value chain (LVVC) for PPR and ND for Sembabule district in Uganda. A gender-sensitive livestock vaccine value chain (LVVC) analysis is used to identify bottlenecks in the entire system, and specifically places where women’s participation is low. A gendered LVVC analysis identifies all stakeholders, systems and processes that would impact men and women smallholders’ individual and collective opportunities. Livestock value chain interventions have been used to understand actors in livestock production systems and opportunities for improvement [19] but a gendered analysis is rarely employed in order to increase vaccine accessibility and adoption by women smallholder farmers.

This paper explores the institutional context within which the PPR and ND vaccine value chains are shaped and the extent that this context affects women’s participation along the LVVC and benefits from the LVVC. The context is framed around institutional and cultural systems that include laws, policies, social norms, attitudes, and practices which operate at all levels from the household to the communities, and government. Understanding the context is critical to determine the most effective interventions to facilitate vaccines adoption and women’s empowerment.

This paper examines four major aspects of the institutional context within which the visibility and positioning of women along the LVVC can be recognized, analyzed, and addressed before developing interventions. These are:

1. The policy and legislative framework that governs PPR and ND vaccine development, distribution, delivery and use in Sembabule district.
2. The characterization, relations, and gender capacities of actors along the PPR and ND vaccine value chains.
3. Perceptions of LVVC actors on gender equality.
4. Women’s visibility along the PPR and ND vaccine value chains and its enhancement.

## Research methodology

### Research area

**Fig 1:**
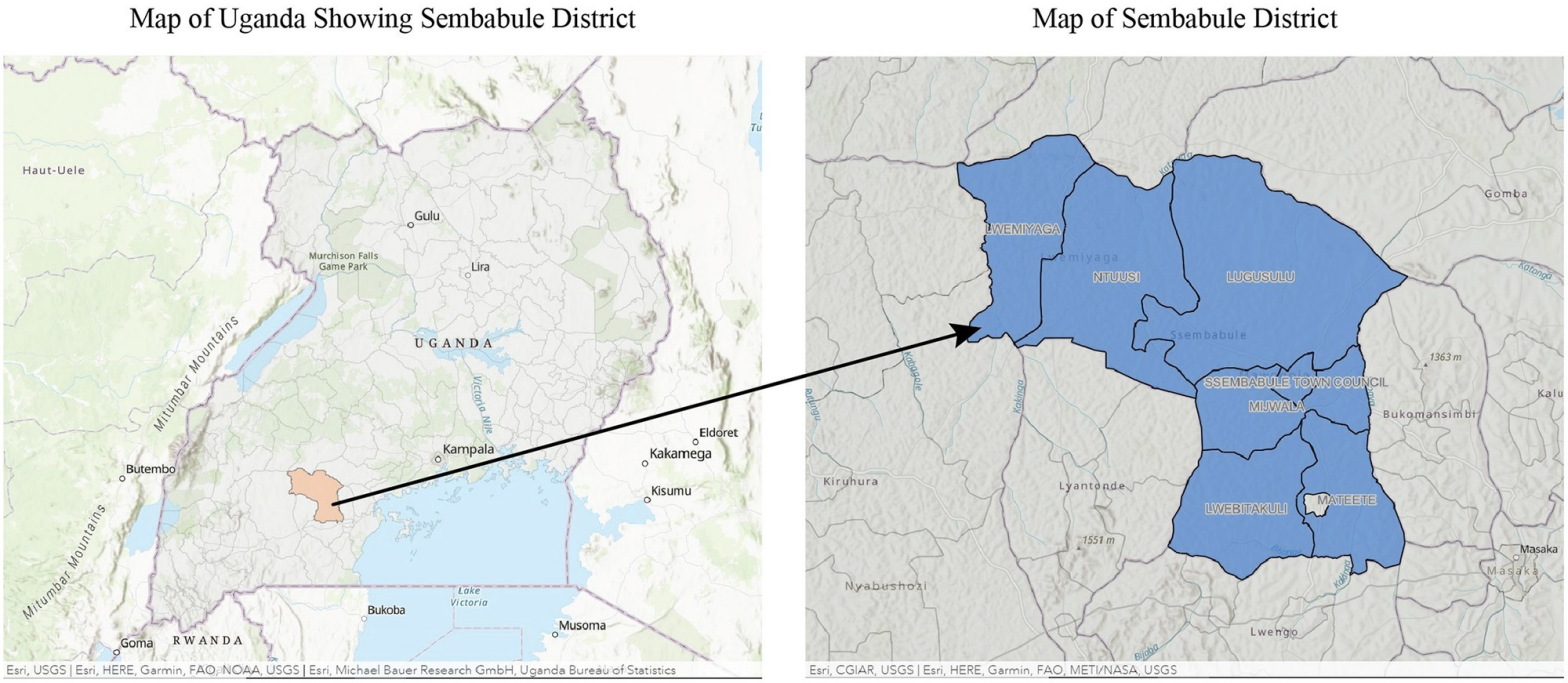
A map of Uganda and Sembabule district. Esri, USGS | Esri, HERE, Garmin, FAO, NOAA, USGS | Esri, Michael Bauer Research GmbH, Uganda Bureau of Statistics.

The research was conducted in Sembabule District of Uganda, which borders Mubende and Kyenjonjo in the Northwest, Mpigi in North, Kiruhura and Lyantonde in the Southwest and Masaka in the East and South. The district has a population of 252,597, 49.9% males and 50.1% females of which 93.2% are rural and 93.3% of the households are engaged in either crop growing or livestock farming [20]. Sembabule District was purposively selected because it is livestock keeping with a history of PPR outbreaks. It consists of two counties, Mawogola and Lwemiyaga: pastoral and mixed farmers. In these two counties, six sub-counties were selected: Mateete, Lugusulu and Mijwala in Mawogola, and Lwemiyiga, Ntutsi and Bulongo in Lwemiyaga County. Fig 1 is a map of the research site. The selection of the sub-counties was done purposively with the assistance of the district production and marketing department to ensure representation of the livelihood categories of the sub-counties, namely purely pastoral, mixed (both crop growing and livestock farming) and semi-urban sub-counties of Sembabule district and the social, cultural and gender dynamics representative of Uganda.

### Study methods

This study employed the qualitative research methods of focus group discussions (FGDs), key informant interviews (KIIs), stakeholder meetings, focus meals (FMs) and jar voices to engage the community and the identified critical partners (stakeholders) along the LVVC. A literature review and expert consultations were carried out to provide a better understanding of the legal and policy environment affecting and influencing the LVVC actors. These methods were employed by the project team which comprised of 5 researchers and 3 graduate students, following a training on the research methodologies and tools for the study. The critical partners were selected by the project team, based on the data that emerged from the institutional and power mapping done by the stakeholders. In addition, a desk review focused on gray literature within the livestock department was conducted to inform the policy and legal framework that governs vaccine development, distribution, delivery, and use. Table 1 presents the tools used in the study.

**Table 1:** Tools used and sample size.

Tools used	Nº of events	Nº of participants			
		Male	Female	Male and female (Mix)	Total
Stakeholder meetings	2	35	20	0	55
Focus groups	20	80	134	0	214
Key Informant Interviews	20	11	9	0	20
Focus Meals	4	0	0	82	82
Jar voices	8	0	0	72	72

### Stakeholder meetings

Stakeholder meetings comprising of 30 (21 males and 9 females) people at national level and 25 (14 males and 11 females) people at district level were first carried out to provide stakeholder analysis, institutional mapping, and power mapping. At the district level, women group leaders from the sub-counties were invited to participate in the stakeholder meeting. Outcome mapping (OM), a qualitative participatory process that allows different stakeholders to collaborate in a systems analysis was used to map and track critical changes, before interventions, in the cultural practices, organizational systems, institutional and governance policies, and the progress of stakeholders towards the goal of women’s empowerment in the livestock vaccine value chain. Participants in the national stakeholder meeting included vaccine regulators, manufacturers, distributors, deliverers, and end users, while those at the district stakeholder meeting comprised of critical partners representing the study sub-counties including the sub-county technical political leaders, women and men chicken and goat farmers, agrovet owners and the district technical veterinary, animal health, gender and animal production teams, and political and administrative representatives from the local government. The OM tool helped to identify LVVC stakeholders and their formal and informal interactions. Through a facilitated process, stakeholders worked collaboratively to physically map out their roles and interconnections in the LVVC, support mechanisms, as well as existing systems including analyzing their current limitations and gaps, challenges, and barriers that they face (both systemic and programmatic). The stakeholders identified challenges and opportunities for women’s participation, engagement, and ability to influence legal and governance structures within the LVVC. The engagements aimed to identify the key actors at the various nodes of the PPR and ND LVVC, to map their interactions, to identify their gender capacities, and the barriers and opportunities that affect women’s participation in and benefit from the LVVC. Institutional mapping is a tool to identify the different nodes in the LVVC, stakeholder roles, and their interactions. Also, the differences between the LVVC for the two diseases were noted. Power mapping is a hands-on tool for visualization, discussion, and analysis of the power of different actors in the LVVC. The women farmers who keep chicken and goats were engaged in deciding who has the power and/or influence and the reasons for and effects of the power differences. They ranked the relative power of each stakeholder by assigning a number from 0 (no power) to 5 (most power). The stakeholders were asked to assess the gender sensitivity and gender dynamics of the key stakeholder institutions along the LVVC using the gender equality continuum tool. The ensuing discussions elicited the gender dimensions of the LVVC, the positioning of women in the stakeholder institutions vis a vis decision-making opportunity, the visibility of women along the LVVC and how it can be enhanced.

### Focus Groups Discussions (FGDs)

Twenty FGDs of up to 2 hours each were conducted. The FGDs were based on the USAID Five Domains of Gender Analysis Framework [21], and focused on barriers, opportunities, and strategies for improving women’s entry, participation in and benefit from the PPR and ND vaccine value chains in Uganda. They dealt with both the customary and legal systems and structures, policies and regulations as well as institutional practices, and on tracing the patterns of power and decision-making at the institutional and community level. Each FGD consisted of a homogenous group of 8–12 women or men. These were community members from the sample sub-counties and different VVC stakeholders representing the different VVC nodes, at both the district and national level.

### Key informant interviews (KIIs)

Twenty KIIs were conducted with Ministry of Agriculture, Animal Industry and Fisheries extension workers, veterinarians, and vaccinators, as well as agrovet owners and attendants, feed store owners and workers, and goat and chicken farmers. The KII were semi-structured guides for either farmers or other LVVC actors, addressing:

1. Farmer knowledge about chicken and goats’ diseases.
2. Gender and age disaggregated access to, control over and benefits from resources.
3. Government policies and activities that affect vaccination.
4. Women’s roles and opportunities to increase benefits from LVVC.
5. Policy and legal matters at national and subnational levels that affect the LVVC.

### Focus meals

Focus meals are impromptu focus groups around a meal with randomly selected participants found in a semi-public setting (near a restaurant or market). A free meal was provided at a random restaurant in a trading center of each sub-county as an incentive for people to share their stories and ideas. Group discussions took place over lunch and took 45–60 minutes. These groups were open to all community members of different genders, making space for those who otherwise may not have participated in the study. Focus meals were used to collect data on knowledge, attitudes, and practices/behavior of farmers about chicken and goat diseases (clinical signs, traditional knowledge cause), disease transmission, prevention and vaccination and gender dimensions hindering women from fully participating and benefitting from the LVVC.

### Jar voices

Jar voices were set up to capture people’s opinions in transit. They were done anonymously for both men and women to collect their views about the gendered ownership of livestock, and participation in and constraints to vaccination of animals. Simple questions were written on flip charts and hung on walls of consented drug shops for a day, then patrons were invited to write their answers and place them in a jar. The answers were collected and replaced with a fresh set of questions the following day. Jar voices helped to capture a community’s identity and voice in real time and space. Jar voices is a very effective tool for capturing voices of people who rarely participate in community gatherings, whose voices are smothered or are rarely selected by their community leaders but can be found in these spaces; the outside or lonely voice. In many patriarchal communities, women belong in this category.

### The gender equality continuum tool

The Gender Equality Continuum Tool (GECT) is used to assess gender sensitivity and responsiveness of different stakeholders in the LVVC. The tool, designed originally for the USAID ASSIST project, helps individuals or groups categorize their gender awareness in behavior and policies and then create a path to achieving transformation of gender relations. The GECT provides a snapshot of the position of each stakeholder, to facilitate discussion moving stakeholders towards gender transformative status, which addresses power inequities between women, men, girls, boys, and non- binary gender groups at the highest levels. This allows for a) a critical examination of inequalities and gender roles b) support and create an enabling environment for gender equality c) promote the relative position of women, girls, and marginalized groups, including transforming underlying social structures, policies, and social norms and d) work to abandon the binary nature of gender [22].

Using the GECT, stakeholders were asked to identify their position along the continuum from gender blind to transformative, and post responses on a chart. The two main categories were gender blind which ignores gender issues and dynamics affecting men and women in the LVVC; and gender aware which examines and addresses gender considerations. Gender aware institutions were further divided into three types: i) exploitative: takes advantage of gender inequalities and stereotypes in the LVVC; ii) accommodating: works around existing gender differences and inequalities in the LVVC; iii) transformative: strengthens or creates systems that support gender equality.

### Gender sensitive model for LVVC analysis

**Fig 2:**
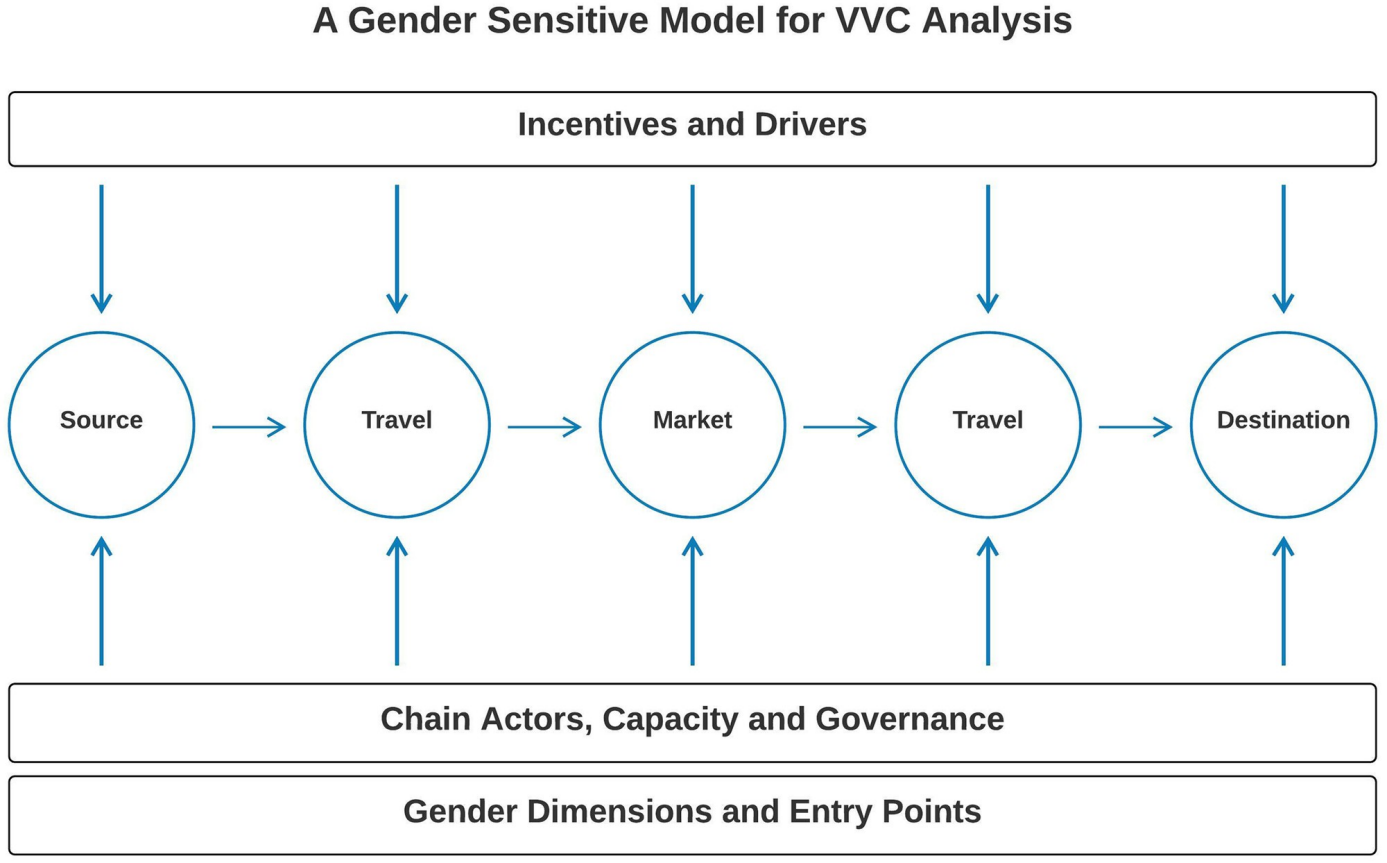
A gender sensitive model for VVC analysis.

Most LVVCs ordinarily demonstrate a linearity, with the value chain framework showing how a vaccine moves physically from the producer to the consumer, increasing in value with the nodes reflecting different actors along the chain. This is usually made up of several actors from manufacturer to distributor, deliverer, and end user as final destination of the vaccine [23, 24]. We present a gender-sensitive model for LVVC analysis (Fig 1). This model was used to identify bottlenecks in the LVVC system, and specifically places where women’s participation is low, allowing strategic interventions for women’s inclusion and promotion of gender equality. The supply chain runs from vaccine manufacturing, through distribution, and delivery all the way to the livestock farmer/end user including the policy and regulatory context [23, 25]. This model considers the chain actors and their capacities, incentives, and drivers, and examines the gender dimensions and entry points (Fig 2). The LVVC analysis helps identify and enact improvements to the regulatory environment and promote systemic transformation of those gender norms that harm families, communities, and nations. We used a gendered LVVC analysis to identify all stakeholders, systems and processes that would impact men and women smallholders’ individual and collective opportunities. This gendered analysis can increase vaccine accessibility and adoption by women, with its follow-on family health benefits and empowerment.

### Data analysis

Data analysis included daily reviews of all data to identify and triangulate key findings. Data collected through key informant interviews, focus group discussions, and focus meals were audio recorded and transcribed verbatim in the local language (Luganda and/or Runyankore) and then translated into English for coding and analysis. Inductive coding of FGD transcripts were compared and contrasted and a comprehensive code book of thematic codes was developed for further data summation and analysis. The coding was based on the five domains of gender analysis, as well as different frameworks such as the gender empowerment framework, chain empowerment matrix, Harvard analytical frameworks, Caroline Moser gender roles framework. The domains and code matrix are provided separately as a supplement. Content analysis was used to examine patterns and interpret meaning [26]. Extracts and quotations were used as examples.

### Ethical approval

Ethical approval for human subjects’ research was obtained locally in Uganda (country clearance Uganda National Council for Science and Technology #RESCLEAR/014March 2019; ethical approval via Makerere University College of Humanities and Social Sciences, Research Ethics Committee #MAKSS REC 03.19.274) and Tufts University Social Behavioral & Educational Research Institutional Review Board (#1907033) prior to commencement of research. Written informed consent was obtained from every participant. For illiterate participants, a thump print was obtained in lieu of a signature.

## Results

### Policy and legislative framework governing livestock vaccine development, distribution, and delivery

According to the director of livestock production in Sembabule district, and other participants at the national stakeholder meeting, current livestock vaccine development, distribution, delivery, and use operate within the broad macroeconomic policies of liberalization and privatization which the Government of Uganda (GoU) initiated in the 1980s as reforms to improve the economic performance of the country. These “Structural Adjustment” policies (SAPs) were augmented by other support policy frameworks, such as decentralization, civil service reform, and good governance, which resulted in a substantial reduction in public sector involvement in the livestock sector and abolition of subsidies on farm inputs like vaccines.

Prior to these policies, the government would provide free extension services, and heavily subsidized inputs like drugs, vaccines, and equipment for farmers. Following Structural Adjustment, the GoU relieved itself of the responsibility of livestock disease control and left it to the farmer. Key informants (veterinarians and animal production officers) revealed that the only notifiable diseases that the Government retained responsibility for are rinderpest, foot and mouth disease (FMD), contagious bovine pleuropneumonia, avian influenza (AI) and rabies. Protection from these five diseases is regarded as a “public good” because they spread so easily, and the diagnosis, control of any outbreak is handled by the Government and its relevant agencies. Rinderpest has since been officially eradicated worldwide but it is retained on the list just in case it re-emerges. For these diseases, the Government procures vaccines (apart from AI where culling is preferred to vaccination) and funds the vaccination processes. Rabies affects all domestic animals except avian species. Nonetheless, the animal rabies vaccine that Government procures is for use in domestic dogs and cats only and is supplied and administered free of charge. Both ND and PPR vaccines are under the jurisdiction of the private sector which must operate within prescribed national laws and guidelines. However, in the past years, Government has intermittently procured and distributed PPR vaccine mainly from the Kenya Veterinary Vaccine Production Institute (KEVEVAPI) in Nairobi, Kenya at no cost. Although this provided some relief especially during outbreak periods, it is inconsistent and unreliable.

Uganda mainly relies on imported vaccines to control most of the vaccinable livestock diseases. The LVVC national stakeholder engagement revealed that there are some private vaccine manufacturing companies in Uganda with established businesses including BRENTEC which manufactures a Newcastle Disease (ND) vaccine. BRENTEC manufactures both thermostable and thermolabile ND vaccines in packages of 100 and 500 doses and is actively engaged in vaccine distribution down to community level in different parts of Eastern Uganda. Imported ND vaccine mainly comes from China, Italy, Spain, and Israel usually in 1,000 dose packages. Although the cost of locally manufactured vaccine is almost the same as that of the imported, the local vaccine has an advantage over the imported one in that it comes with a device for dispensing and the smaller packages of 100 and 500 doses make it more user friendly to the smallholder local chicken keepers. Table 2 shows the cost of ND vaccine as shared by one of the distributors.

**Table 2:** Cost of new castle disease vaccine.

Vaccine pack (doses/vial)	Cost/vial (UGShs)	Cost/dose (UGShs)
100	3,500	35
500	5,000	10
1,000	7,000–17,000 (depending on country of origin)	7–17 (depending on country of origin)

Source: Personal communication with a distributor.

Members of the regulatory bodies clarified that both the importation and development of livestock vaccines are governed by the National Drug Authority (NDA) under the National Drug Policy and Authority (NDPA) Act CAP 206 of 1993. The NDA is the Government agency mandated by law to deal with the development and regulation of human and livestock drugs and vaccines in the country and to ensure the availability of efficacious and cost-effective vaccines and other drugs to the human and animal population in Uganda. Under the Act, no person or body shall import drugs and vaccines into Uganda without a license from the NDA and no person shall import or sell any drug including vaccines, unless it appears in the national formulary. Prior to issuing an import license, the NDA ascertains that the facility in which the vaccines are manufactured, complies with the internationally accepted Good Manufacturing Practice Guidelines as adopted by the NDA.

Drugs listed under the First, Second and Third Schedules of the Act are Class A, B and C restricted drugs that can only be sold by authorized sellers and in licensed premises. In the case of vaccines, the licensing process must verify the presence of a stable and efficient cold chain. The sale of restricted drugs can be undertaken by licensed persons, under the immediate supervision of a pharmacist only. For a person to be licensed to sell veterinary drugs and vaccines, they must possess a minimum qualification of at least a certificate in veterinary practice from a recognized institution and operate from licensed premises. This means that veterinary drug and vaccine sales require a pharmacist, and the licensed veterinarian must also attain a pharmacist license or employ one, at additional expense.

The delivery of livestock health products and services is regulated by the Animal Diseases Act (CAP 38), the Veterinary Surgeons Act (CAP 277) and the National Drug Policy and Authority (NDPA) Act CAP 206 of 1993. The NDA is charged with the responsibility of implementing the National Drug Policy and of regulating the development and operations of pharmacies in the country. It approves the national list of essential drugs, registers drugs, issues licenses for the wholesale and retail trade of drugs and controls the importation and sale of pharmaceuticals.

Information obtained from key informant interviews and stakeholder meetings, indicated that there are no officially recognized Community Animal Health Workers (CAHWs) in Sembabule district. This is because it is assumed that Sembabule district has enough qualified veterinary workers and therefore there is no need to permit CAHWs who are most times without any veterinary qualifications. In some parts of Uganda, for instance in the Karamoja Sub-region, the government allows and even supports CAHWs. This is because Karamoja is considered a hard-to-reach area with no/or inadequate social amenities which make it less attractive to qualified veterinary service providers. The CAHWs include community vaccinators who provide vaccination services for livestock, including poultry. Their operations, however, are outside of the legal and policy framework, given their absence of recognition in both the Veterinary Surgeons’ Act and the National Drug Policy and Act. However, they remain professionally accountable and are supervised by the veterinary authority in their area of operation.

### Characterization, relations, and gender capacities of the PPR and ND vaccine value chain actors

From the outcome mapping exercise, many actors were identified as being involved in the LVVC from manufacturing, distribution, delivery and use of both PPR and ND vaccines. Institutional mapping and stakeholder analysis at the national and district level identified policy makers and regulators, vaccine manufacturers, vaccine importers and distributors, vaccine deliverers and vaccine users as the five broad categories of actors. According to the OM process, these are the critical partners in the PPR and ND LVVC who have the power to improve vaccine delivery and women’s participation and benefits from it. The gender capacities of institutions were measured using the GECT by stakeholder self-reports on the existence of documentation, supportive systems, political will, and structures which address the existing gender differences and inequalities. Most stakeholders (64%-n = 9) identified their organization‘s behaviors as gender accommodating. Nearly 28% (n = 4) of the institutions self-identified as exploitative. No stakeholders identified their institution as gender blind and only one organization identified self-identified as gender transformative(Table 3).

**Table 3:** Classification of organizations along the gender equality continuum using the GECT.

Place on Gender Continuum	Type of Organization in Category	No. of Organizations
**Gender Blind**	0	0
*Organizations ignore the influence of gender and deny its effects*		
**Gender Exploitative**	Agrovet/ veterinary pharmacies and shops	4
*Organizations take advantage of gender inequalities to their benefit*		
**Gender Accommodating**	Government ministries, departments and agencies, veterinarians’ organizations, pharmaceutical companies [vaccine manufacturers, importers and distributors], research	9
*Organizations identify and then accommodate or work around gender inequalities*		
**Gender Transformative**	Academia	1
*Organizations critically examine and challenge gender inequalities attempting to make substantive change*		
**Total**		**14**

### Policy makers and regulators

**Fig 3:**
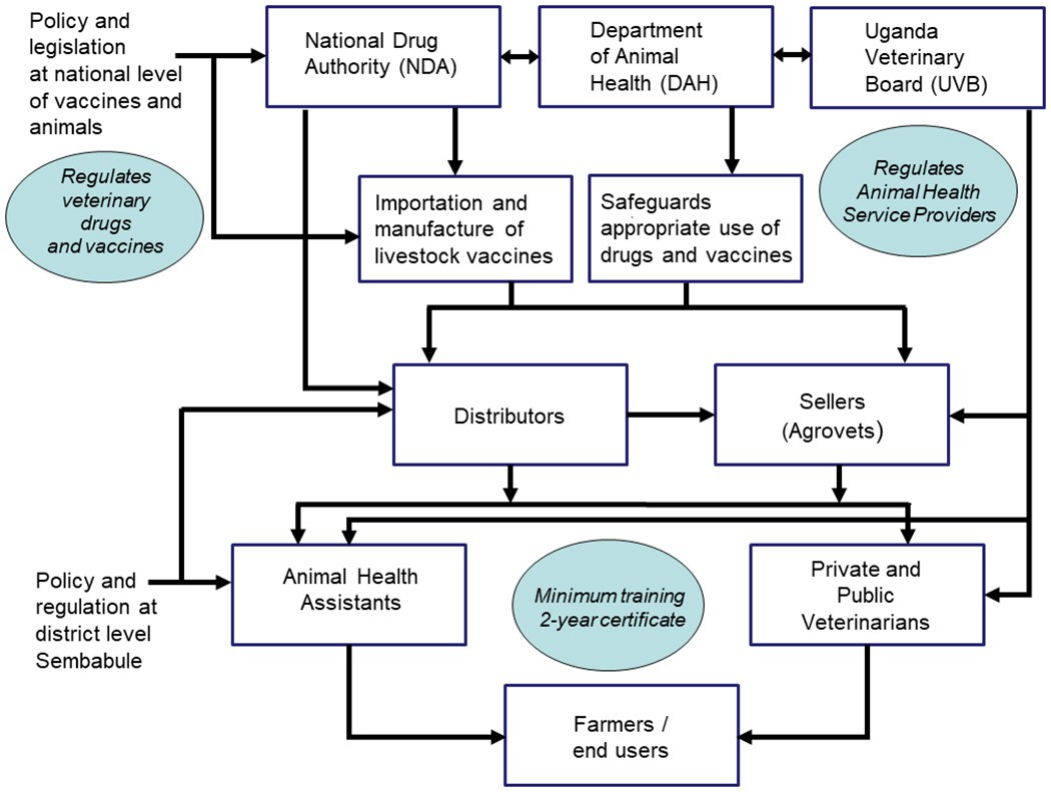
Regulation of veterinary, vaccines, animals, and animal health service providers in Uganda.

The policy makers and regulators work at the national and district level (Fig 3). At the national level, they are the MAAIF, NDA, Uganda Veterinary Board (UVB) and Uganda Veterinary Association (UVA). MAAIF and NDA develop and implement policies and regulations that govern livestock vaccines and vaccination, monitor, and regulate veterinary service delivery, issue licenses for authorized dealers in vaccines, and ensure efficacy and effectiveness of the vaccines. MAAIF is the government institution responsible for animal health and wellbeing and under specific circumstances imports and distributes the PPR vaccines through district veterinary services.

The Uganda Veterinary Board is a professional regulatory body established by an Act of Parliament (The Veterinary Surgeons Act 1958, Cap 277). Its major mandate is to ensure that animal health services are offered by qualified, registered, and licensed veterinary professionals under their regulatory supervision. To do this, the Board runs several activities including, but not limited to, registration of professionals and their premises of practice; establishment of standards for training, practice, and professional conduct of veterinary professionals; and provision of guidance and support for continuing professional development programs and community education. The UVA, on the other hand, is an umbrella organization that promotes and protects the interests of veterinarians in Uganda and collaborates with other stakeholders in the animal industry. UVA’s mission is to foster socio-economic development and improved livelihoods of the veterinarians through optimization of animal resources.

The Department of Animal Health (DAH) of the Directorate of Animal Resources (DAR) under the Ministry of Agriculture, Animal Industry and Fisheries (MAAIF) is the public agency entrusted with ensuring the health and wellbeing of livestock and other animals in the country through provision of satisfactory animal health care and safeguarding the appropriate use of drugs and vaccines for animal health. It does this through a network of field staff supervised by the District Veterinary Officer (DVO). There are also Private Veterinary Practitioners (PVP), who work under and report to the DVOs of the respective districts. They are required to regularly report on their veterinary activities including clinical work, vaccinations, surgical interventions to the DVO of the district in question. The professional conduct of all veterinary practitioners in the country is overseen by a statutory body known as the Uganda Veterinary Board (UVB) which is appointed by the Minister responsible for Animal Industry, and with which they must be registered in order to be allowed to practice.

Gender responsive budgeting, to improve targeting and service delivery for equal benefit and opportunities for both women and men, is a key aspect of gender integration in government programs given that it is encouraged from the national level through the Office of the Prime Minister. Efforts to integrate gender equality at the planning, budgeting, programming, monitoring and evaluation within the department programs at district level were mentioned as the government’s effort toward gender integration. Integration entailed analysing and addressing gender related challenges which limit achievement of gender equality, such as incorporating programs which reflect women’s specific needs, for instance provision for a crèche for nursing female staff. One way to promote gender integration at the district level was to attach monetary incentives for districts that performed well in integrating gender in their programs. Local government staff were trained in gender awareness. There were gender focal points at the district level, who were majorly women. This is because “gender concerns” are still considered a woman’s domain.

In spite of these positive steps, it was evident that these local government efforts were only pursued when external agencies, such as the national government or funding partners, purposed to “push” the gender agenda. Although the departmental staff had received gender awareness training, application of the knowledge and skills gained was lacking. The deployment of the veterinarians in their work assignments was found to be “gender blind” as observed by some participants in the stakeholder meetings.

The Uganda Veterinary Board (UVB) and the Uganda Veterinary Association (UVA) tended to be gender accommodative since they worked around existing gender differences and inequalities instead of transforming the gender relations. They ensured provision of promotion opportunities for qualified and competent female workers and deployed female workers based on their personal circumstances, such as nursing mothers. For instance, nursing mothers were not posted to field stations that are away from their homes, whenever such an opportunity was available. However, they lacked clear documentation of practices which promote gender equity or equality and transformative processes that challenge the deep-rooted causes of gender inequalities.

### Vaccine manufacturers and distributors

At the national stakeholder meeting, and in focus group discussions, the distributors were mainly categorized as the relatively big or medium sized pharmaceutical companies. They include BRENTEC, ERAM, Biyinzika and MTK companies. They are mainly importers of vaccines, who store them at relatively large distribution points in Kampala and their centers in major towns which represent regions such as the East, West, North and South. For instance, Masaka is the nearest southern big distribution center which serves its neighboring districts including Sembabule district. These centers distribute vaccines and other drugs to agrovet or veterinary pharmacies and shops in these districts. Some of these companies have a dual role of distribution and manufacturing and are legally allowed to reconstitute and repackage the vaccines to smaller doses/vials following guidelines from the regulators. For example, ND vaccine is officially sold in 1,000 dose vials. Some companies repackage it into 50 to 100 doses to enable affordability for smallholder farmers. However, due to the continuous demand by farmers and limited government supply of PPR vaccines (PPR is not among the 5 priority diseases for government control), sometimes the vaccines are smuggled in from Kenya and are administered covertly. BRENTEC and ERAM are key LVVC actors who manufacture and distribute ND vaccines along with drugs and other animal products through their medium and large distributors, who transport and deliver them to wholesale outlets in various districts of the country.

Using the GECT [22], the vaccine manufacturing and distributor companies rated themselves as being gender accommodative (work around gender inequalities, rather than address them). They did not have specific gender policies in place and the livestock vaccine manufacturing and importing sector tended to be male dominated. Both BRENTEC and ERAM claimed the existence of institutional gender responsive practices within their organizations, without providing evidence. Both companies had women in key leadership positions, gained through merit.

They observed that the NDPA Act is silent on gender issues that may affect the manufacturing and importation and distribution of veterinary health care products including vaccines. The stakeholders reported that they also lack policies that would encourage gender mainstreaming (incorporate gender perspectives) in their functions.

The main vaccine distributors in Kampala and Jinja reported that they were gender aware (they examine and address gender inequalities). As an example, one of the distributor companies claimed that their management had a plan to institute a gender policy to address the current staff recruitment, deployment, promotion, and remuneration which currently do not bear any gender considerations.

### Vaccine deliverers

Vaccine deliverers ranged from relatively large, organized firms with multiple outlets across the country to small individual or private companies operated by one person, local governments (districts), and public veterinary service providers at the district and sub-county level who also operate private businesses as individuals, individual private veterinarians, veterinary drug shop owners, to community vaccinators. Male *boda boda* riders who use private motorcycles for public transportation of people and commodities, including vaccines, were considered key actors in the vaccine delivery node. Gender equality considerations were observed only among the public vaccine deliverers, namely the local governments. An example, a nursing center has been constructed at the district site to address the needs of the nursing female district staff. In this way, a more enabling environment for the nursing mother is created. This, however, may not necessarily address the unequal power imbalances between the women and men.

### Vaccine end users

Vaccine end users were mainly commercial farmers, small holder and medium holder men and women farmers, either as individual farmer households or as farmer groups, and poultry breeding companies.

There are many women farmer groups involved in chicken rearing in Sembabule, a few of which involve men. Men are mainly involved in goat farming given government encouragement for commercialization of goats and as a growing main income source for many poor households. At both the household and group level, men/husbands were identified to play a key role in buying the vaccines from the distribution points as well as in vaccinating livestock. Smallholder farmers also highlighted the joint partnership they had with the large scale/commercial farmers to purchase vaccines from Kampala city: *“we jointly ask the farmers with many goats (large scale) to buy the PPR vaccine since it is only sold in packages of 50 and 100 doses then when the vaccines are delivered*, *we are also given [vaccines] for our few goats”* [FGD-24, female goat farmer, Mijwara sub-county].

In Sembabule district, PPR and ND vaccination coverage is very low (estimated at less than 10% by one of the veterinary officers). This is attributed to inadequate supply of vaccines but also the stigma some farmers attach to vaccination. They think it is a ploy to harm their livestock. As a consequence, the concept of “herd immunity” cannot be achieved. Herd immunity is said to have been achieved when most of a population is immune to an infectious disease which provides protection to those who are not immune to the disease (not vaccinated). Women farmers claim that their stock are not prioritized as much as stock belonging to men. They feel that their small flocks are deliberately not targeted for vaccination by the private sector or government. “*Gavumenti negyema ente omumwanya ogu konka tihine orikutekateka aha mbuzi ne enkooko nobuzakufa omubwingyi”*, translated”*the government massively vaccinates cattle in this area*, *but no one thinks about goats or chickens even though they die in larger numbers*” one woman said. The women feel that there are gendered politics in livestock disease control systems that need to be transformed. Even though this cannot be confirmed, there is evidence that livestock services and product delivery systems are male dominated, and women lack access to these services [12]. Some factors like vaccine cost, distances to vaccination points for goats, and lack of or limited access to vaccination information all appear to favor men achieving higher vaccination rates.

Two categories, namely the vaccine transporters and research institutions were mentioned as LVVC actors, although they were not given attention as critical partners in the involvement of the project, since they were considered as less influential/powerful. The vaccine transport delivery chain, at the national level, included air transport for vaccine importation, cold chain vehicles owned and used by government, public buses, and private company vehicles. At the sub-national level, private company vehicles, public vehicles and *boda bodas*, featured at the vaccine transportation node. Government-imported vaccines are transported by air to Entebbe International airport and using cold chain vehicles owned by commercial clearing agents, the vaccines are delivered to the government central stores in Kampala or Entebbe from where they are distributed to districts using government-owned cold chain vehicles or picked from the stored by district local government-provided means of transport.

**Fig 4:**
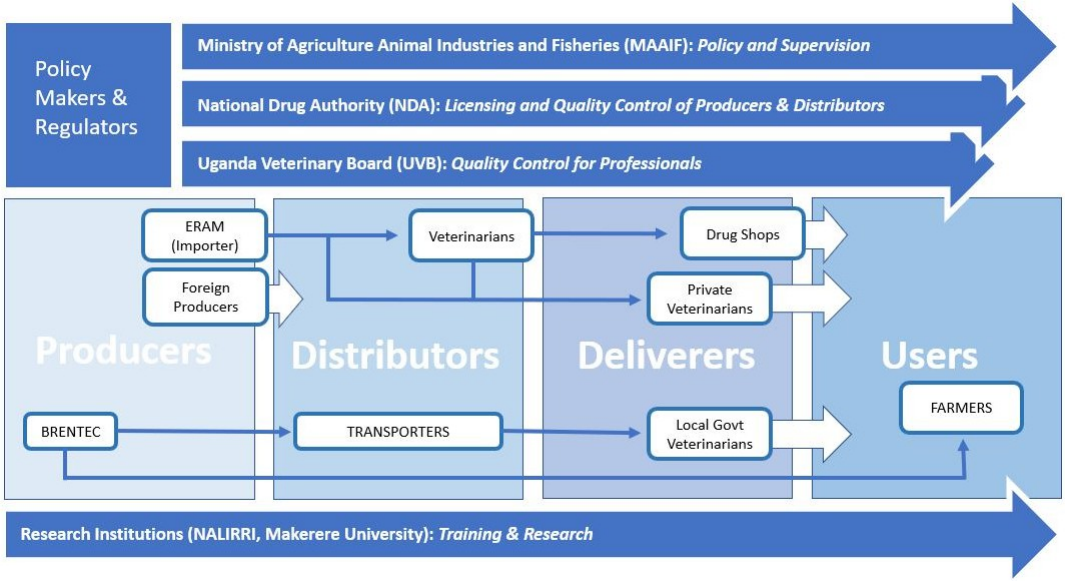
Private sector-led ND vaccine value chain.

Makerere University and the National Animal Genetic Centre and Data Bank (NAGRIC) & DB were at the national stakeholder meeting, representing research and training institutions. They identified their roles as conducting research and training on livestock vaccines and vaccination. They claimed to be gender aware even though they did not have any gender-related institutional policies or practices around their research and training on vaccines and vaccination. Through institutional mapping and power mapping, the stakeholders, both at the district and national level, viewed the private sector-led ND vaccine value chain, (purely driven by the private sector, with no government involvement), as constituting a complex web of actors, all of them operating in the district with very minimal, if any, interaction. Fig 4 shows a LVVC map drawn by stakeholders showing their perceptions of existing structures and interlinkages between the key chain actors of a typical ND vaccine value chain. It also depicts the actors at policy and operational level both at the national and sub-national level.

Apart from the policy makers and regulators who have distinct roles of policy making, supervision of veterinary service delivery, and licensing of lawful operators, the rest of the vaccine chain actors operate almost at all levels and with different actors of the ND vaccine value chain. These relations, under the private-led LVVC, are less linear, hence fluid and sometimes multiple, with an actor playing multiple roles and relating with different actors. Vaccine manufacturers, for instance, indicated that they operate almost at all levels including delivering directly to farmers especially when there is a new product on the market which they want to introduce to the end users. Vaccine manufacturers also indicated flexibility of offering training directly to farmers on vaccines and vaccine use if well organized in order to open new markets.

Vaccine users, as indicated in Fig 4, interact with all vaccine chain actors, since the ND vaccine is a private good. There was no mechanism in place (public or private sector led) to coordinate all the actors. The district production office indicated that they were planning to register all private sector players and support them to have leadership in place. Although NDA as regulators are supposed to be active at the district level, their presence in districts including Sembabule is lacking, and this has potential to affect the quality of service to the end user as quoted by one district official: *“NDA does not have the capacity to monitor and enforce the guidelines*, *they are largely missing in the district*, *and have no arrangements with the district production department to support them”*.

**Fig 5:**
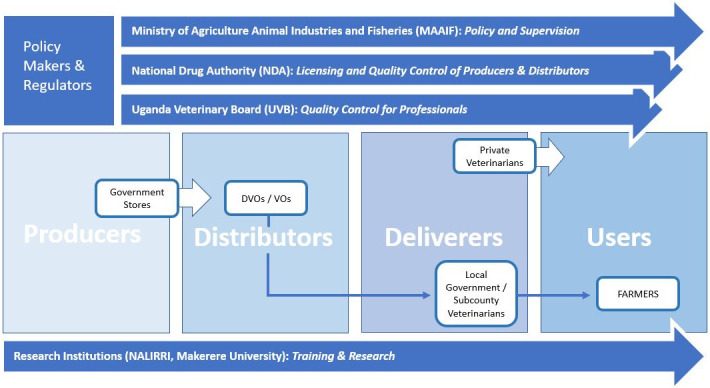
Typical government-led PPR vaccine value chain.

The PPR vaccine supply chain is linear, as shown in Fig 5.

Although PPR vaccine supply is not deemed a public good, its importation, storage, distribution, and delivery is government-led. Government supplies PPR vaccines free of charge (sometimes as donation by Food and Agriculture Organization of the United Nations (FAO)) mostly to the Karamoja sub-region where PPR outbreaks are more common due to cross border livestock movement from South Sudan and Northwestern Kenya. Due to other priorities, the PPR vaccines procured are not enough to cover sufficient goats and sheep to achieve herd immunity. Sembabule and other districts in the “cattle corridor” of Uganda have also experienced outbreaks and have benefitted from government supplied vaccines. Before then, the national decision makers widely believed that PPR disease was a problem of the Karamoja sub-region as portrayed by one of the Sembabule district officers: *“…it is misunderstood to be a Karamoja sub-regional disease by government”*. This is because the Government used to only supply PPR vaccines to the Karamoja sub-region, and not to the other districts like Sembabule. When PPR started spreading to other districts from 2009 onwards, the government started considering them as well for vaccine supply.

The difference between the PPR and ND vaccine value chains lies in the fact that PPR is a relatively new disease in the country and is perceived to cause greater socio-economic harm and therefore the government is compelled to spend public money towards its management, even though it is not among the five priority diseases it is mandated to control.

The perception that PPR causes greater socioeconomic harm than ND is subjective but is based on varied public responses to outbreaks of the two diseases. Whereas ND has been in existence in the country since pre-colonial times, no documented serious government efforts are known to have ever been put in place to control it, even though it kills thousands of birds every year. ND control is a private affair that is handled by farmers individually, with the source of vaccine being the private sector. Hence, it attracts many actors with a profit-making motive. Yet with PPR, which was first diagnosed in the country as recently as 2007, the government makes efforts to procure vaccines for free distribution to affected districts. The two varied approaches by the government creates the impression that PPR is of greater importance than ND.

### Perceptions of LVVC actors on gender equality

Both men and women were involved in goat and chicken rearing. Even when the goats and chicken belonged to the men, some men expected the women in their households to rear them. Women and men farmers’ attitudes and beliefs about women’s involvement in vaccination varied. Some of the farmers believed that it was the responsibility of men farmers and male veterinary extension workers to vaccinate their livestock, since vaccination by injection required strength and technical competence, which were more likely to be possessed by men. This is reinforced by gender norms as demonstrated by the following quotes: *“Generally it is the men that are responsible for that kind of work at home”* [female farmer (R14), FGD—Lugusulu women chicken farmers]. *“Naturally*, *women cannot vaccinate in our setting and that has been for a long time now”* [LC Chairperson, FGD—Lugusulu women chicken farmers].

The majority of men and women smallholder farmers and veterinary extension agents had negative attitudes and beliefs about the involvement of women veterinary extension agents in vaccination of goats and cattle and in the treatment of diseased livestock. They argued that the nature of the job could not allow for women’s engagement. The job itself requires physical strength, especially if it entails administration of injection, willingness, and commitment to travel to and work on farms, many of which could be only accessed by motorcycles.

The female agents seldom rode for fear of being stigmatized and falling victims of motorcycle related accidents which occur due to the rough terrain. Most respondents noted that men were more involved in vaccination and treatment of livestock, depending on experience and willingness to engage in field work, which includes vaccinating livestock.

The extension agents, both male and female, during key informant interviews and focus meals, observed that many qualified female veterinarians preferred to do lighter work, such as being dispensers at vaccine and drug outlets, while men conducted field work, including vaccination. One female paravet mentioned: *“…but what I have discovered*, *women who are brave enough to go to the field are very few*. *If you scan around in this area*, *you can’t find another female vet*. *She finishes school and aims at sitting in a drug shop and will tell me I don’t want to go to the field”* [Female paravet, Focus Meal].

They claimed that the qualified female agents were more inclined to obtain convenient work which was “soft” and “clean” and located in urban centers. They felt that the female agents tended to have fear, lacked self-confidence and willingness to engage in field work. They observed that their claims were backed up by the large numbers of female veterinary professionals who were currently being employed at the distributor outlets in urban areas. *“It is due to the nature of work which requires a person to be energetic*, *continuous movements*. *Secondly*, *the employers*, *e*.*g*. *distributors*, *won’t give a woman to drive a route for a whole month distributing drugs*. *For instance*, *this morning I was called to go to farm 8km away then rush back here to attend to you*, *then after here I have to go to another farm that is 10km away*. *Sometimes we go hungry and on empty stomachs*. *These are things that discourage women*. *In fact most of the women I studied with are at container village selling drugs*. *According to me*, *women are most distributed in the selling and dispensing of drugs”* [Male government veterinarian].

They noted that some female extension workers suffered from stigma and perceived blame from others when they engaged in certain kind of work which is perceived as men’s work, including vaccination of goats and cows, and motorcycling for field work: *“You can vaccinate a farmer’s animals when there is failure*, *they won’t blame the vaccine but the woman who will have vaccinated*. *If it’s a man*, *he won’t be blamed*. *Women fear to ride motorcycles or do field work*. *They prefer to sit at drug shops”* [Female Veterinary Assistant/paravet, Sembabule Veterinary drug shop].

Several farmers preferred male veterinarians to female veterinarians to attend to their goats. This is largely due to cultural issues, which affect their beliefs and perceptions. Men are considered strong and resilient, while women are the weaker ones. In one of the discussions during a focus meal, all the farmer participants agreed with a respondent who said that: *“It is a man [that is preferred] because the animal might be powerful*, *and it is a man to handle it and also inject it”*.

One of the male veterinary extension agents however observed that some female extension agents were as competent as their male counterparts: *“Doctor*, *that question you have asked*, *it is both sided because sometimes I go with this female colleague of mine and farmers wonder*, *eeh a woman treats animals they have never seen it*. *But indeed*, *you find she’s better than some men if she has handled animals well*, *used drugs and dosages well and the animal recovers quickly which is possible for the man to fail to treat the animal—so it falls both sides*. *Our people have that thinking of long ago”* [Male para-vet, Focus Meal 29].

### Women’s participation and benefits along the PPR and ND vaccine value chain

On the positioning and visibility of women in the PPR and ND vaccine value chain (LVVC), data from the FGDs show that women were largely involved at the end user level, mostly as farmers and to a lesser extent, as poultry breeders/suppliers (end user). Very few women and men farmers vaccinated their goats and chickens. For those who did, the women farmers vaccinated against ND. In some cases, the women farmers who did the actual vaccination themselves, or whose husbands or hired labor performed the actual vaccination, sometimes reared already vaccinated chicken to ease the rearing burden of very young chickens.

In all the focus group discussions of women farmers, the women farmers ranked themselves, both as individuals and as groups, as the most important stakeholder in the LVVC as the owners of the chickens and goats, as small-scale chicken breeders and suppliers, and as the chicken rearers themselves.

At the end user node, there was low vaccine demand from women smallholder farmers. This was attributed to the cost and packaging of the vaccine and lack of knowledge relating to the need for vaccines for local goats and backyard chicken, which the women farmers largely reared. The available vaccines tended to be expensive and were packaged in large dose vials (100), which were unfavorable for smallholder farmers, given the small number of livestock that they reared. To increase their sales, the agrovet shops resorted to reconstitute vaccines for farmers so that they could buy what they could afford, according to the number of chickens that they reared, raising questions of vaccine quality and efficacy. In addition, farmers lacked cold chain carriers to keep the vaccine cold.

The focus group discussions, key informant interviews and focus meals revealed that overall the vaccine supply for both PPR and ND vaccines in Sembabule district was limited at the marketing, distribution, and delivery chain nodes. The PPR vaccine on the Ugandan market was not readily available. Only one male government veterinarian was sometimes able to import the PPR vaccine from Kenya and avail it for use by the goat farmers in Sembabule District. There were no agrovet or veterinary drug shops which stocked PPR vaccines in Sembabule district, while only a few agrovet/veterinary drug shops stocked ND vaccines that were imported largely from Indonesia. The livestock drug shop owners attributed this to lack of cold chain facilities, including refrigerators and cooler boxes, their dependence on electricity supply for their refrigerators which was erratic, thereby compromising the quality of the vaccines, and low farmer demand for vaccines.

At the production and manufacturing node, the prohibitive cost of starting up and operating a vaccine manufacturing plant and capital-intensive requirements to set up vaccine distribution outlets hindered women participation, as owners. Production of biological vaccines requires technical competence through advanced training, which few women veterinarians had. This is partly attributed to their expected role in society. A woman, after completing her first degree, is expected to get married and start bearing children by the age of 30. Thereafter, she takes care of her family and home, which makes it difficult for her to undertake advanced training, which largely requires her to spend some months away.

At the distributor node, women were more visible as employees of the manufacturing firms and as dispensers or attendants/salesclerks at distributor outlets, attendants at agrovet/veterinary drug shops in some of the Sembabule trading centers, and in small- to large-scale vet drug distributor outlets in large towns, including Masaka and Kampala city. The attendants at agrovet/veterinary drug shops were not required to be trained in animal health service delivery. A few female veterinary extension agents, including one female veterinarian, were largely poultry vaccinators at the household and community level.

Only one woman was a shareholder (part owner) of a large distributor outlet. In some cases, the women attendants were wives of the drug shop owners. The attendants sold/dispensed veterinary drugs and to a lesser extent, vaccines and provided vaccine information and advice to the farmers, including how to use vaccines and drugs. The attendant at the Kinyenya drug shop, for instance, observed that all the attendants of this particular drug shop were always women. Apart from being owners of the drug shop, men also were engaged as hired labor for tasks that required physical strength, for instance carrying the heavy things to and from the shop store. The licensing process which enables veterinarians to acquire licenses for animal pharmacies or drug shops tended to disfavor women since it requires staying away from home for a number of days, as she pursues the license in Kampala, as a key female informant observes: *“ideally by the time you get this license [from Kampala] you have lost yourself to so much or the possibility of not getting all you needed because of money shortage*. *These challenges are the same for men and women*, *only challenge [for women] is late night travels and sexual harassment”* [KII, Agrovet 5, Female private veterinarian, Bulongo parish].

### Enhancement of participation and benefits of women in the LVVC

Through FGDs and KIIs with women and men farmers, community leaders, agrovet shop dealers and animal health service providers, several barriers and opportunities were identified, and recommendations were made to enhance women participation in and benefit from the LVVC.

At the delivery level, some participants felt that the female drug shop attendants can potentially be involved in vaccination and in field extension work, if their capacity can be built and gender barriers (including motorcycle riding, stigma, fear and blame due to social and cultural norms, negative perceptions, and attitudes) that they encounter are overcome. Some respondents observed that parents could play an important role by encouraging their daughters to study veterinary sciences.

A majority of women farmers envisioned themselves being involved in the LVVC at the end user and distributor level and as chicken suppliers. They felt if they had access to financial support and credit, inputs, and other capacity building support, they could own agrovet/vet drug stores as a means to generate more income and access to more knowledge and skills in poultry management. As indicated below by one of the participants in a FGD, when asked about women’s participation in the LVVC: *“I see women only fitting in the farming section alone because they stay home mostly*. *Women farmers see themselves in the agrovet business mostly as well as the young chicks selling; this in the long run will help them to learn more and acquire skills when it comes to the poultry business”* [Woman chicken farmer, FGD—Lugusulu women chicken farmers].

In summary, the stakeholders identified the barriers to increased participation and benefits from the LVVC as lack of education, experience, entrepreneurial skills, and capital to operate poultry enterprises. Women’s involvement in poultry and livestock agrovet/vet enterprises was also constrained by having too many responsibilities at home, which did not allow them freedom to engage in activities outside of their homes.

Recommendations to enhance women participation and benefits at the marketing and distributor chain level included boosting their capital, supporting them to set up outlets that are closer to the communities, strengthening their capacity to engage with communities, and building their self-esteem. The female extension workers could be provided and supported to efficiently operate a cold chain as part of a starter kit to facilitate their businesses and to increase vaccine supplies.

At the end user lever, recommendations included transforming the women groups into viable and profitable commodity groups, utilizing the commodity groups for awareness raising and serving as a community role model, increasing chickens and goat stock, and establishing a mechanism for supervising and supporting beneficiaries.

## Discussion

The control and eradication of livestock diseases is only possible with involvement of all stakeholders along the LVVC, from vaccine manufacturers and distributors, regulators, deliverers, and women smallholder farmers who are mainly at the end-user level. According to Rathod et al. (2019), global eradication of rinderpest was only possible due to the roles played by all stakeholders, including livestock owners [27]. This was achieved with minimal understanding of gender dynamics in livestock keeping because cattle are considered an animal for men. Disease control for goats and chickens must include the women who care for them every day, and rely on them to feed, clothe and educate their families. A holistic and sustainable model that focuses on systemic transformational change within the animal health sector to value women’s contribution, and support their empowerment is essential.

Vaccine uptake is a complex process which requires buy-in from men and women farmers, veterinary departments, county/district and national governments, and vaccine producers. Vaccine uptake ultimately depends on the social context and must respond appropriately to the power dynamics in the household, community and across the entire livestock vaccine value chain. We have to recognize intra-household dynamics, control over resources and who decides what. Gender roles and relations in the households intersect with positions, relationships and responsibilities which must be understood to create truly transformative projects that raise the position of women relative to men. The gender blind history of livestock development projects has all too often resulted in increasing the workload of women without empowering them. Coupling interventions that enhance the equity of the social environment in the LVVC and technical components such as training, and provision of the cold chain can enhance women’s instrumental agency, and lead to better outcomes for women, men, their families and communities.

The crucial role played by rural women in livestock production and management must be more widely recognized and their participation should not be limited to the end user level. Creating more entry spaces for women along the LVVC and opportunities that foster a vertical shift of women along the LVVC from end users of the vaccines and veterinary drug shop attendants to becoming women entrepreneurs and decision-makers at the various nodes of the chain is critical for improved livestock productivity in Sembabule district of Uganda. However, the predominantly private nature of the livestock LVVC for the management of PPR and ND in Sembabule district disproportionately affects the women chain actors compared to the men. The high cost of the vaccine, dependence on market forces, and the bureaucratic rigors in licensure which predispose women chain actors to an insecure environment are examples of institutional barriers which hinder them from competing favorably in the market space. This is in agreement with some studies [28].

This study shows that both PPR and ND vaccine access is critical for increased vaccine adoption and women empowerment. Nevertheless, most women are clustered at the end user level. Providing women access to vaccines and/or other resources, markets and technologies does not automatically translate into women’s control over them or their benefits, or into their social acceptance of new roles and opportunities. For this translation to occur at an institutional level, a two-pronged approach is required. First, a visible mechanism for collaboration among the chain actors to recognize and address the barriers and opportunities for women is needed. Secondly, there is need to engage and support women and men to transform gender relations, so they recognize and address the harmful power imbalances, for increased livestock productivity and improved household well-being. This is in agreement with several studies [29–32].

Successful collaborations would entail leadership and financial resources from the District Production and Marketing Office to bring together the LVVC actors to jointly address the identified barriers to women participation and benefits in the LVVC. Formation of partnerships between distributors and users, between large or medium pharmaceutical companies, the veterinary drug outlets and the extension workers can be nurtured. The LVVC actors could lobby and advocate for a policy change that will include PPR on the list of public good diseases to avert the devastating impact of the PPR outbreaks in Sembabule district, and other parts of the country.

With increased gender awareness and capacity building of the collaborating institutional actors, the collaborative effort would contribute towards changing the negative perceptions of the chain actors on gender equality and facilitate the shift of the institutions’ gender practices from tokenism to gender transformation. This would include taking on deliberate actions which support the women’s efforts towards improved household livestock productivity and women empowerment, which otherwise the women on their own, would not have been able to address. Also, ensuring technical and human resource gaps in the animal health service delivery sector are addressed would further contribute to the benefits of the collaborative effort. A gendered perspective and analysis on barriers to livestock production and disease prevention (i.e. mitigation, adaptation, policy development) decision-making needs to be applied. Understanding the different barriers women smallholder livestock farmers face as opposed to men is critical, as it informs policy makers in developing gender-sensitive and more informed programmes to enhance livestock farmers in general and women’s welfare in particular. Considering gender differences in livestock management and production and reflecting them into livestock projects and policies is critical. Quisumbing et al (2015) explain that sometimes the unique barriers men and women experience in value chains result from gendered customary norms, roles, and stereotypes [33].

To enhance women’s empowerment efforts, gender transformative approaches that embrace joint partnerships and networks which benefit women and women groups are critical to enhance the emerging intrinsic agency that some women farmer groups identified. Effective GTA requires political commitment to changing the status quo, and allocation of budget for resources for trained staff and their transportation, and adequate time for reflection and change. Or else gender inequalities persist or even increase after many development interventions. A holistic and sustainable model that focuses on systemic transformational change and empowerment for the women smallholder farmers is essential. The female commodity farmer groups and networks could be linked to the national farmers group in order to leverage on resources from both groups.

## Conclusion

To increase vaccine adoption, improve livestock productivity and empowerment among small scale women farmers in Sembabule district requires going beyond provision of vaccine-related and gender equality training, infrastructural and technical support to the poultry and goat women and men farmers and other chain actors. This will require building and nurturing an intentional and visible mechanism that fosters gender transformative collaborative efforts among the LVVC actors and builds viable gender-transformed women groups and networks to enhance women participation in and benefit from the livestock vaccine value chain. Use of a gender transformative approach will enable the LVVC actors to direct their efforts to addressing the institutional barriers which hinder women’s empowerment.

Enhanced positioning and visibility of women in the LVVC can occur through a systemic engagement of different critical partners, and a recognition of the responsibilities and roles of women under the initial stewardship and coordination of the District Production and Marketing Office. Including women farmers when defining and shaping the potential entry points is critical, given their central roles as critical partners in the LVVC. Viable farmer-distributor networks can be established, promoted and nurtured for increased vaccine accessibility, knowledge about vaccines and livestock disease prevention and control, and increased vaccine adoption.

## Supporting information

S1 Table Code matrix. (DOCX)

S2 Table Focus group discussion coding example. (DOCX)

S1 File (DOCX)
