# Supplementary material for: Breaking institutional barriers to enhance women’s participation in and benefit from the Peste des Petits Ruminants and Newcastle Disease vaccine value chains for Sembabule district of Uganda
Source: PLoS One. 2022 Oct 13;17(10):e0270518. doi: 10.1371/journal.pone.0270518 (PMC9560069; doi:10.1371/journal.pone.0270518)
Supplement: S1 Table — (DOCX) [file pone.0270518.s001.docx]

| **Domains** | **Subthemes** | **Codes** |
| --- | --- | --- |
| 1.  Laws, Policies, Regulations and Institutional Practices (The Chain Empowerment matrix) |  | Gender / identity of government veterinary workers |
|  | Institutional practices | Influence of institutional practices |
|  |  | Cooperation |
|  |  | Private sector |
|  |  | Religious practices |
|  |  | Support/aid programs |
|  |  | Government engagement |
|  | Policy interventions | Intervention (policy, programs, etc.) |
|  | Veterinary policy | Veterinary role in vaccine administration/VVC |
|  |  | Practices/policies to include female veterinarians in school |
|  | Vaccine Policy | Limited vaccines registered by law |
|  | Accountability of government | Local/public administration |
|  | Regulations | Disposal of dead animals |
|  | Barriers |  |
|  | Opportunities | Cooperation |
| 2.  Access to and Control over Assets and Resources (including income, employment, and assets such as land) (Harvard Analytical Frame-work, Gender Empowerment matrix) | Access | Access to resources |
|  |  | Access to finances |
|  |  | Access to opportunity |
|  |  | Access to education |
|  |  | Access to social assets |
|  |  | Access to human capital |
|  |  | Access to physical environment |
|  |  | Access to personal assets |
|  |  | Access to livestock |
|  |  | Access to technology |
|  |  | Access to leadership positions for women |
|  | Barriers to access | Drivers license |
|  |  | Transportation / moto not comfortable for women |
|  |  | Livestock mortality as a barrier to economic success |
|  | Control | Control over human capital (skills one has, knowledge and skills) |
|  |  | Control over resources |
|  |  | Control over social assets (networking, groups) |
|  |  | Control over finances |
|  |  | Control and decision making over family |
|  |  | Control and decision making over goats |
|  |  | Control over personal assets |
|  |  | Control over physical environment |
|  |  | Control over opportunities |
|  | Barriers to control |  |
|  | Opportunities |  |
| 3.  Gender Roles, Responsibilities and Time Use (Caroline Moser Gender Roles Frame-work) |  | Dependence of women on men |
|  |  | Ability to multitask |
|  |  | Women and men spend their time differently |
|  |  | Gender roles |
|  | Allies | Male allies |
|  | Time availability | Time availability for women |
|  | Animal type ownership | Chicken |
|  |  | Goat |
|  | Biology | Pregnancy |
|  |  | Access of women to agrovet positions |
|  |  | Difficulty to adopt roles that are not usual for this gender |
|  | Barriers | Job opportunities |
|  | Opportunities | Prioritizing girls vs. boys |
|  |  | Gender-based opportunity |
| 4.  Cultural Norms and Beliefs (Force-field analysis) |  | Women are weak/have no control |
|  |  | Hanging cultural norms |
|  |  | Impact of misogyny on women |
|  |  | Community norms and beliefs |
|  |  | Consequences of norms and beliefs |
|  |  | Judgement of women based on decisions to use resources |
|  |  | Men's perceptions of women buying drugs |
|  |  | Influence of support program on family culture and gender norms |
|  | Relationships | Power struggle |
|  |  | Marital conflict |
|  |  | Comparing and measuring success of wives |
|  |  | Relationship/communication between husband and wife |
|  | Barriers |  |
|  | Opportunities |  |
|  |  | Gender norms and becoming female importer |
|  |  | Female role models encourage more female involvement |
|  |  | Prioritizing education for girls vs. boys |
|  |  | Female Farmers cultural norms and use of vaccines and raise animals |
|  |  | Auto marginalization |
|  |  | Women as animal keeper |
|  |  | Girls preference in term of education |
| 5.  Patterns of Power and Decision-making (Household decision-making models-Unitary and collective/cooperative conflict models) | Decision roles | Women's decision making and control over family |
|  |  | Decision making power to choose to become female veterinarian |
|  |  | Opinion about why men allow women to have control and decision-making power |
|  |  | Strategies to influence husband's control and decision making |
|  |  | Influence of communication on men’s control and decision making |
|  |  | Barrier to women having control and decision-making power |
|  |  | Influence of culture and beliefs on men's control and decision making |
|  |  | Strategies to influence husband’s control and decision making |
| 6. Education, training, and skills | Training | Benefits of training |
|  |  | Trained community members |
|  |  | Benefits of training women to provide veterinary care |
|  |  | Access to training for women |
|  |  | Training on vaccination |
|  |  | SMS and technology training |
|  |  | Empowerment training |
|  |  | Training of advisors |
|  | Education | Access to education for women |
|  |  | Benefits of education/training women |
|  |  | Education for girls |
|  | Knowledge | Lack of knowledge on vaccine and vaccination |
|  |  | Lack of Knowledge on livestock health and management |
|  | Barriers | Access to information - barrier |
|  |  | Barriers to women participating in training and education |
|  | Opportunities | Opportunities for training to improve access and control over assets |
|  | Sensitization |  |
|  | Group organization | Group structure and organization |
|  |  | Culture of community groups based on gender structure |
|  |  | Group formation |
|  |  | Benefits of participation in groups |
|  |  | Building group capitol |
|  |  | Challenges for women's groups |
|  |  | Group activities |
|  |  | Aspirations for participation in groups |
|  |  | Goat ownership facilitated by groups |
|  |  | Consequences to group membership |
|  |  | Influence of group membership on family culture |
|  |  | Control over group membership |
|  |  | Negative consequence of group membership |
| 7. Livestock ownership, disease and prevention | Vaccination | Goat vaccination |
|  |  | Cold chain |
|  |  | Access to goat vaccines |
|  |  | Access to drugs / vaccine |
|  |  | Vaccine packaging |
|  |  | Type of vaccines |
|  |  | Cost of vaccine |
|  |  | Source of vaccines |
|  |  | Lack of vaccines |
|  |  | Access form women to VVC |
|  | Veterinary services | Access to veterinary services |
|  |  | Gender/identity of veterinarian |
|  |  | Distance to livestock health services |
|  |  | Lack of veterinary extension services |
|  |  | Equipment (storage transport) |
|  | Disease management | Herbal and other treatment modes |
|  |  | Poultry disease management |
|  |  | Goat disease management |
|  |  | Causes and symptoms of poultry diseases |
|  |  | Causes and symptoms of goat diseases |
|  |  | Alternative NCD/ CCPP treatment |
|  | Ownership of livestock | Goat ownership facilitated by groups |
|  |  | Goat ownership |
|  |  | Challenges to raising goats |
|  |  | Barriers to raising goats - poverty |
|  |  | Goat breeds |
|  |  | Barriers to goat ownership for women |
|  |  | Goat husbandry and management practices |
|  |  | calendar for chicken mortality |
|  |  | Benefits to raising chickens |
|  |  | Control over livestock |
|  |  | Barriers to access and control over livestock |
|  |  | Opportunities for training to improve access and control over assets |
|  |  | Mortality of goats |
|  |  | Mortality of chickens |
| 8. Empowerment |  | Desire for empowerment |
|  |  | Women's empowerment |
|  |  | Priorities of empowered women |
|  |  | Benefits to empowering women |
|  |  | Barriers to empowerment for women - cultural beliefs |
|  |  | Opportunities to improve respect of women |
|  |  | Control over self |
|  |  | Self esteem / women’s confidence |
|  | Barriers | lack of role models |
|  | Opportunities | training |
