# Supplementary material for: Breaking institutional barriers to enhance women’s participation in and benefit from the Peste des Petits Ruminants and Newcastle Disease vaccine value chains for Sembabule district of Uganda
Source: PLoS One. 2022 Oct 13;17(10):e0270518. doi: 10.1371/journal.pone.0270518 (PMC9560069; doi:10.1371/journal.pone.0270518)
Supplement: S2 Table — (DOCX) [file pone.0270518.s002.docx]

**FOCUS GROUP DISCUSSION 9**: NTUSI WOMEN GOAT KEEPERS/ coding sample

**Domains 2 and 3**: Access to and Control over Assets and Resources (for example Land) Gender Roles, Responsibilities and Time use were discussed.

| **Number of participants: 24 Women**. |
| --- |
| **Duration: 3hrs & 15 minutes (approximately)** |
| **Facilitator**: Dr Elizabeth Kyewalabye |
| **Note taker:** Ms. Lillian Tukahirwa |

The above two members from SheVax++ Project started by introducing themselves to the women’s’ group. A prayer was followed from one the participants. The purpose of the meeting was explained and then the consent form was read to the participants. They were requested to select one person from the group to sign on their behalf which they accepted.

The women were asked how many goats they had and it was observed that the range was from 5 to 35 goats per household.

The following questions were administered to the participants and the responses were as follows:

| **Facilitator** | **Respondents** | **Codes** |
| --- | --- | --- |
| **Elizabeth:** How is land access in this area like? | **Rs:** All respondents say that they have access to land. They are allowed to use it for production. | **Land access** |
| **Elizabeth**: How about control over land? | **R1**: We don’t have control over land.  **R2 (Widow)**: I have control over since I don’t have a husband  **R3**: I have control over the land since I don’t have a husband | **Control over land** |
| **Elizabeth:** Who Benefits from land? | **Rs:** The whole family benefits but men take the high percentage. For example when harvest is done, men take a big share after selling the produce | **Benefiting from the land** |
| **Elizabeth:** Do you easily access to Water?  **Elizabeth:** How about access and control of Crops (Cash & Subsistence)? For example: Beans, Maize, cassava, matooke, Groundnuts, peas and coffee. | **Rs:** We have access to water only that we suffer during dry seasons where we are forced to move long distances and sometimes we are stopped from fetching water from the owners of the dams. Sometimes we pay money, each jerrican 1000= to the boda boda guys who fetch water.  **Rs:** Yes both men and women control the crops with men taking control on most especially coffee and motooke and leave the rest to women.  **Rs:** Men take 60% and women take 40% despite the fact that men take only coffee and matooke. These two crops yield more money than the rest of the other crops.  **Rs:** When some crops are sold, some money is used to pay school fees for children. | **Access to water**  **Seasonality of water access**  **Cost of water**  **Access and control of crops**  **Use of benefits** |
| **Elizabeth:** How about access and control of trees? (Ovacado, Oranges, Guavas, Jackfruits and mangos): | **Rs:** We have access to trees but our men take the control. The trees benefit the whole family in providing food and sometimes money for school fees when sold. | **Control over trees** |
| **Elizabeth:** How about access and control over livestock? (Chicken, eggs, manure, and goats) | **Rs:** We have access to livestock, for the control, it depends on the owner of the livestock. If the livestock is for the man, he is the one with the control. If it is for the woman, she has some control but not full control. Even if this woman wants to sell this livestock she has to first ask for permission from the man. Women can eat eggs, and use manure for gardens.  **Rs:** When the livestock are sick; the man doesn’t take the responsibility of treating them, even if he has his. It is the woman to look for the Veterinary Doctor to treat these livestock and pay.  **Rs:** Women lack information of market and when the woman asks the man to go and look for the market, this man ends up cheating her in terms of selling price. This man takes the goat to the market or goes and looks for the buyer and they negotiate the price before coming to pick it. For example; he sells the goat at 200,000= when he comes back, he tells the woman that I sold it at 150,000= and also asks for some money from that 150,000= he has given her after making the difference (enjawulo) of 50,000=.  **Rs:** It is in few cases where the man pays for the treatment of these livestock. So, in terms of who benefits more from these livestock, men benefit 60% and women 40%.  **Rs**: Cattle are owned by men and women access and benefit from milk. | **Access and control over livestock**  **Women empowerment**  **Women access to veterinary care**  **Payment for veterinary care**  **Access to information**  **Goat marketing**  **Benefits from goats**  **Women negotiation power**  **Men’s integrity**  **Payment livestock treatment**  **Access to benefits from livestock** |
| **Elizabeth:** Vaccination of livestock, who is responsible?  **Elizabeth:** How do you graze your goats? | **Rs:** The owner of the livestock does the vaccination of his/her livestock. **Rs:** We normally vaccinate when these livestock are already sick which leads to death of some livestock.  Whether they understand the difference between vaccination and treatment:  **Rs:** We know the difference, vaccination is before they fall sick and treatment is after they have fallen sick. When asked how many women do the vaccination of their livestock; out of 25 women only 7 women vaccinated their livestock from kipumpilu (NCD), Diarrhoea, Kihaha (CCPP), Kibumba (Liver problems) and okukorora (cough). Women were asked why they don’t vaccinate:  **Rs:** We don’t have money to buy the vaccines, also Vet Doctors are not available but we are willing to participate in the vaccination in case we are trained. Most of the vaccination exercise is done by men.  **R1:** We tie them in the bush in the morning and bring them back in the evening  **R2:** others said that they use free range system. | **Role in vaccination**  **Vaccination calendar**  **Knowledge of vaccination**  **Affordability of vaccines**  **Availability of veterinary doctors**  **Feeding of goats** |
| **Elizabeth:** How is labour input in goat keeping? | **Rs:** Women offer more labour: ranked with 3 out of 5, and men 1out of 5, children and house helpers (workers) shared one. Each getting 1/2. This labour include feeding, watering and cleaning the goat house. | **Gender Roles** |
| **Elizabeth:** What are your sources of cash/income? | **R1:** Women have groups from where they get cash  **R2:** Women get cash after selling their livestock and some produce. | **Sources of finance** |
| **Elizabeth:** Where do women obtain information from? | **Rs:** We obtain information from our men, friends, children and neighbours. | **Information access** |
| **Elizabeth:** Where and how do you sell your goats?  **Elizabeth:** What is the level of education in this community? | **Rs:** Men are the ones who look for the markets and also determine the price of their livestock since for them.  **Rs:** Most of the women are illiterate. Men are the ones with access to education and they have control over education. | **Market access**  **Literacy levels**  **Access and control over education** |
